# Ablation Analysis to Select Wearable Sensors for Classifying Standing, Walking, and Running †

**DOI:** 10.3390/s21010194

**Published:** 2020-12-30

**Authors:** Sarah Gonzalez, Paul Stegall, Harvey Edwards, Leia Stirling, Ho Chit Siu

**Affiliations:** 1 Department of Aeronautics and Astronautics, Massachusetts Institute of Technology, 77 Massachusetts Avenue, Cambridge, MA 02139, USA; stegall@mit.edu; 2 Lincoln Laboratory, Massachusetts Institute of Technology, 244 Wood Street, Lexington, MA 02421-6426, USA; harvey.edwards@ll.mit.edu (H.E.); hochit.siu@ll.mit.edu (H.C.S.); 3 Department of Industrial and Operations Engineering, Robotics Institute, University of Michigan, 1205 Beal Avenue, Ann Arbor, MI 48109, USA; leias@umich.edu

**Keywords:** human activity recognition, surface electromyography, inertial measurement units, feature selection, wearable sensors

## Abstract

The field of human activity recognition (HAR) often utilizes wearable sensors and machine learning techniques in order to identify the actions of the subject. This paper considers the activity recognition of walking and running while using a support vector machine (SVM) that was trained on principal components derived from wearable sensor data. An ablation analysis is performed in order to select the subset of sensors that yield the highest classification accuracy. The paper also compares principal components across trials to inform the similarity of the trials. Five subjects were instructed to perform standing, walking, running, and sprinting on a self-paced treadmill, and the data were recorded while using surface electromyography sensors (sEMGs), inertial measurement units (IMUs), and force plates. When all of the sensors were included, the SVM had over 90% classification accuracy using only the first three principal components of the data with the classes of stand, walk, and run/sprint (combined run and sprint class). It was found that sensors that were placed only on the lower leg produce higher accuracies than sensors placed on the upper leg. There was a small decrease in accuracy when the force plates are ablated, but the difference may not be operationally relevant. Using only accelerometers without sEMGs was shown to decrease the accuracy of the SVM.

## 1. Introduction

Human Activity Recognition (HAR) aims to classify motions with the goal of characterizing the behaviors. HAR has previously been performed while using both wearable and external sensors [1]. External sensors, such as cameras, photometric sensors, and motion capture systems, are common tools in activity monitoring, but they have their drawbacks. Cameras and photometric sensors are commonly used to represent “ground truth” labeling [2], but they are limited by occlusions and shadows from other objects in the environment. Additionally, activities present in a variety of ways when viewed from different angles, so picking the proper view for observation becomes an issue for two-dimensional (2D) systems [3]. Marker-based motion capture systems, where only the three-dimensional (3D) marker coordinates are recorded, have been shown to be more reliable at activity detection than cameras alone, but the system requires a setup that can be quite costly and it can also be affected by occlusions and extraneous reflections [3].

Wearable sensors, such as surface electromyography sensors (sEMGs) and inertial measurement units (IMUs), have also been explored for HAR [4,5]. Walking and running are not only biomechanically different, but they also present differences in EMG activity over a range of fixed gait speeds [6]. The different EMG profiles make EMG useful for observing activity differences on various subjects with self-selected natural gait speeds, where one person’s running speed might be another person’s walking speed. Although, sEMGs are not without their drawbacks. If the primary muscle used for a specific activity has a weakened signal like in cases of patients going through rehabilitation, the sEMG would need to be placed on another, related muscle [7], potentially decreasing the accuracy of the activity classification in situations where the sEMG needs precise placement. If the muscle of interest is in a region with multiple muscles, then sEMG signals might need to be processed in order to separate out confounding signals [7]. Another common wearable sensor is the accelerometer. Although useful on their own, these widely available sensors increase HAR accuracy in combination with other sensors, such as sEMGs [5,8]. Every sensor has its benefits and drawbacks, but it has been shown that sensors can be used in tandem with HAR, and this fusion leads to increased accuracies in recognition of defined activities [9]. Examples of these fusions are using EMGs and IMUs (which contain accelerometers) for HAR for flexion and extension motions of the trunk [10], as well as IMUs and pressure sensors to recognize sitting, standing, walking, and running [11].

Data from sensors can be used in order to classify activities while using supervised machine learning techniques, such as Support Vector Machines (SVMs). The accuracy of an SVM is affected by the features selected and activities defined, as the methods are trained while using these data. Supervised models are trained while using a set of features and known activity labels. In machine learning, a feature is a characteristic of the data collected. Creating a definition of activities and their specific characteristics is one of the largest challenges of HAR [2]. Previous efforts across a range of activities have had accuracies that range from 70 to 95% [5,12,13]. These studies differ in the sensors and features used and the activities detected, leading to the different measured accuracies. It is unclear which features or sensor signals drive accuracy and further effort is needed in order to assess sensitivity and specificity for selected features.

The data and time needed for training will increase with the dimensionality of the system, which can occur by adding additional sensors and features [2]. Dimensionality reduction techniques, such as singular value decomposition (SVD) and principal component analysis (PCA), can be used in order to eliminate noise and reduce the need for large sets of training data [14,15]. When PCA is used for HAR in literature, it is typically used only for feature selection [1,8].

This paper seeks to understand which sensors drive accuracy, sensitivity, and specificity in human activity classification. The categories of “standing”, “walking”, “running”, and “sprinting” were chosen because they are common activities that humans perform. Furthermore, data ablation is used in order to show that sensors on the lower leg produce higher classification accuracies than sensors on the upper leg. Through the ablation analysis, this paper provides the novel contribution of determining which sensors and sensor placements contribute to the classification accuracy and an interpretation of these findings. The subjects performed these motions using a self-paced treadmill, which enables users to select their own walk and run speeds, creating a natural variability within and across subjects. In order to use data that are transformed by PCA to train an SVM, it must be established that these classifications are appropriate for use between subjects and trials. PCA weightings are vectors in a high dimensional space; therefore, to determine that these weightings are similar and can be applied across subjects and trials, the angles between the weighting vectors were analyzed. The effect of the sensor set on classification accuracy will be presented. First, the paper will discuss the methods that were used to conduct the study. Subsequently, the results of the principal component similarity test and accuracies of the SVM training will be presented and discussed. This research informs how wearable sensors can be selected for classifying standing, walking, and running.

## 2. Materials and Methods

### 2.1. Experimental Setup

**Figure 1:**
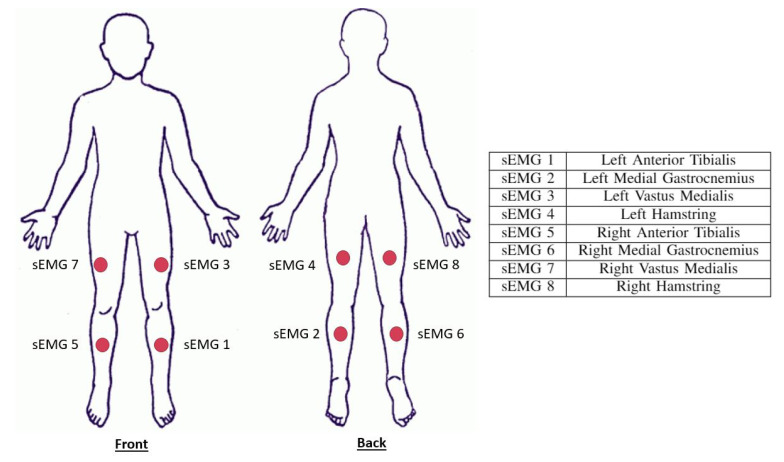
Trigno sensor locations on the subjects. These sensors include both a surface electromyography sensor (sEMG) and an accelerometer

The experiment was performed at the MIT Lincoln Lab Sensorimotor Technology Realization in Immersive Virtual Environments (STRIVE) Center in the Computer Assisted Rehabilitation Environment (CAREN) (Motek Medical, Amsterdam, The Netherlands). The CAREN is a 24-foot diameter virtual-reality dome with a six-degree of freedom platform. The platform had a self-paced, split belt treadmill that was equipped with two force plates, one under each foot. Though these force plates were not wearable sensors, they could reasonably be replaced with force sensitive shoe insoles. For data collection, each subject was outfitted with wireless Trigno sEMG sensors with onboard accelerometers (Delsys Inc., Natick, MA, USA) that were placed precisely on certain muscles (Figure 1). Additionally, each subject was outfitted with motion capture markers, so the Vicon system (Vicon, Oxford, UK) in the CAREN could record the subject’s motion. The marker set used was a modified Plug-in-Gait marker set, where the marker that was set on the upper body was reduced, as the focus of the study is on the lower body. These markers were used for the self-paced treadmill control and they were not used for activity classification.

**Figure 2:**
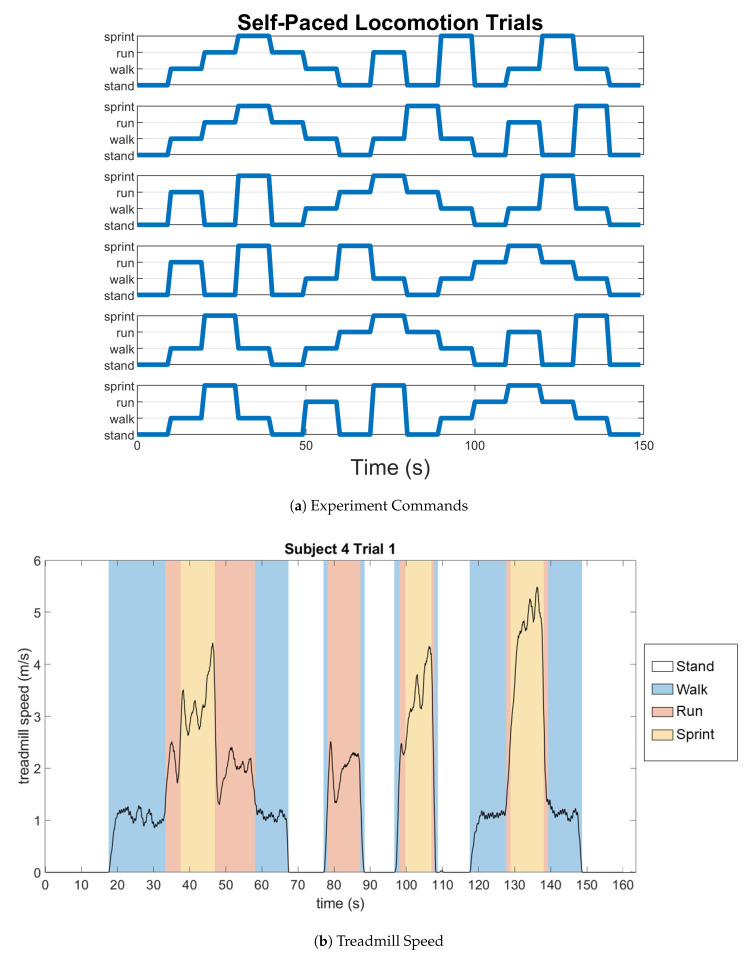
(**a**) The distribution of the commands each subject was given across time. There are six plots, one for each of the six trials. (**b**) Example of the self-paced treadmill speed for the first trial Subject 4 completed, where the commands are reflected in the speed at which the subject was moving. Note that while commands were given for the same duration, the activities are not all performed for the same duration, justifying the decision to label data points by speed instead of commanded activity.

The experiment had five subjects with a mean weight of 69.6 ± 19.9 kg and a mean age of 24.5 ± 4.3 years, all of whom were physically capable of completing all tasks. Subjects performed six trials on a treadmill, where they were given commands of “stand”, “walk“, “run”, and “sprint”, via on-screen text. New commands were issued every ten seconds, and each trial lasted 150 s. The order of the commands was switched for each trial in order to capture a roughly even distribution of transitions between different states (Figure 2). The MIT Committee on the Use of Humans as Experimental Subjects approved the procedure, and all of the subjects gave voluntary, informed, written consent to participate.

### 2.2. Feature Selection

All of the features were extracted while using a sliding window of 0.5 s with 0.30 s overlap, similar to previous work [16,17]. The accelerometer data from all eight sensors were used as features by taking the vector norm of the accelerometer data in each window (ACC). The mean vertical force that was applied to the individual force plates (FP) as well as the standard deviation of the force applied to the force plates were also used as features. The median frequency (MF) of each sEMG sensor was used as a feature. It has been found that the frequency domain is useful in assessing muscle fatigue, which is important, because, as the trials progress, the subject tires, so it is important to ensure that our classification works for all levels of fatigue [18]. The sEMG signal samples in each window were binned by assessing the mean and standard deviation (SD) within that window in order to generate amplitude bin features (H1, H2, H3). Three bins were used for 0 to 1 SD from the mean (H1), 1 to 2 SD from the mean (H2), and >2 SD from the mean (H3). This method was chosen, because histogram features work well in high dimensional space, leading to higher robustness in recognizing activities [19].

In total, there were 44 features used: two force plate means (FPM), two force plate standard deviations (FPSD), eight sets of accelerometer data (ACC), eight sets of median sEMG frequencies (MF), and eight sets of three sEMG bins (H1, H2, H3). All of the features were z-score normalized in order to ensure that all data are on the same scale.

### 2.3. PCA Analysis

PCA is a dimensionality reduction technique that creates new axes for a set of data that maximizes the variance that occurs along the axes. From an m×n set of data, where *m* is the number of observations and *n* is the number of features, PCA creates *n* orthogonal principal components, where component 1 is a vector in the feature space where the data have the most variance and each successive component contains decreasing variance, so component *n* is a vector where the data show the least variance. These components are defined by a n×n set of weights, known as coefficients, for each feature. The coefficients indicate how much each feature is correlated to that particular principal component. Each feature has a corresponding score, where the scores are representations of the data in the space of the PCA coefficients, such that T=XW, where *T* is the original data set, the rows of *X* are the coefficients of each score, and the columns of *W* are the scores of each component [20].

The comparison of PCA weightings between subjects was performed by determining the angle between the orthogonal vector sets. The angle between every combination of trials without repeating combinations and the median of every angle was found. An angle between the PC coefficients can be calculated, because these weightings are the basis of an orthogonal set of vectors. The angle between components can be found while using the dot product of coefficients of the trials being compared. Smaller angles between principal components indicates a greater similarity in the features. Similarity in a matrix comparing two vectors would mean low values on the diagonal. Because PCA weightings indicate variance in either direction of the corresponding vector in feature space, we can take the supplementary angle when differences in vector angles are more than 90 degrees in order to account for the axis being identical, whether it is srepresented as positive or negative. In this study, the supplementary angle was taken for comparisons between likecomponents. In this context, “like-components” refer to the same principal components that originate from different trials and/or subjects (e.g., principal component 1 from trial 1 and principal component 1 from trial 2). Before the supplementary angle was taken, there was a bi-modal distribution that was centered around zero. To more accurately reflect how far from 0° these angles were, the like-components were reported in a range from 0° to 90°. All of the comparisons were done between the first three principal components of each dataset, as they describe approximately 85% of variance of each dataset. These comparisons were performed across all trials and subjects.

### 2.4. Support Vector Machines

A SVM was used in order to classify the PCA data into categories of motion. SVM finds separating hyperplanes, such that data points with different labels are maximally separated. These data can be “classified” by using the side of the hyperplane on which they are located [21]. A SVM was chosen from other supervised learning techniques, because visual inspection and initial observations of the data supported the use of a hyperplane to separate the data into categories. A Gaussian SVM, which uses a Gaussian kernel, was used in order to separate the data into the categories of stand, walk, run, and sprint. Because there was a natural delay between the subject reading the command and then performing it, we were unable to classify actions based on the time that the command was initially displayed. Instead, actions were classified manually (Table 1). For training, speed thresholds were manually set based on the speed that the user was maintaining at each point in time, as determined by an inspection of the treadmill speed across time. This labelling also included the transition regions. For example, if the subject was commanded to transition from standing to running, then the portion of data where the subject was moving with their determined walking speeds (Table 1) was labelled as walking (Figure 2). Subsequently, the class labels that were used for training were filtered with a sixth order Butterworth filter with a cutoff frequency of 50 Hz to smooth changes in speed due to high frequency noise.

### 2.5. Accuracy

The accuracy of the SVM was found while using six-fold cross validation, where whole trials were reserved one at a time for testing. The accuracies for each subject were only calculated within each subject. During the cross-validation, PCA was performed on the data that were reserved for training. The data reserved for testing were transformed into the PCA space of the training data while using the coefficients found; no separate PCA was performed on the testing data. The accuracy was defined as the percentage of data points that were correctly classified by the SVM. Because the main difference between running and sprinting is speed rather than a biomechanical difference, such as presence or lack of double support as in walk vs. run, the accuracy was also computed when prediction confusion between running and sprinting was allowed by grouping running and sprinting labels together. Additionally, the sensitivity and specificity of each set of sensors are reported. Sensitivity is the proportion of correctly-classified data points with respect to the overall data points in that class, and specificity is the the proportion of correctly-classified negative data points versus the number of data points that are truly negative. Positive data is a correct activity classification, while the negative data is an incorrect classification. All of the machine learning and mathematical calculations were completed using MATLAB.

### 2.6. Ablation

**Figure 3:**
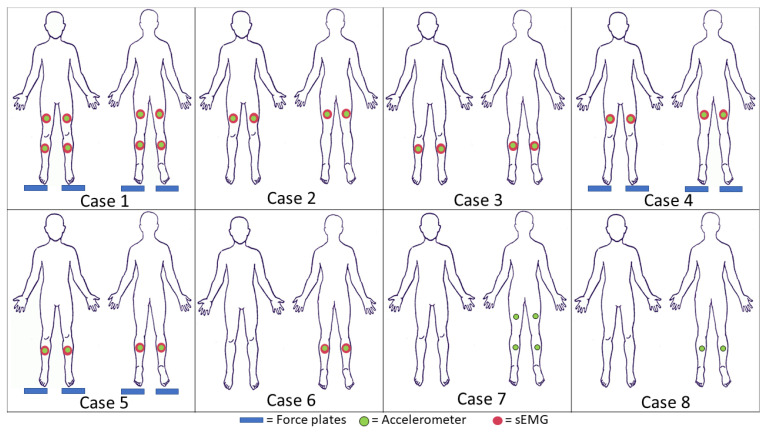
Locations of sensors during ablation. Case 1 includes all sensors. Case 2 includes sensors on the upper leg. Case 3 includes sensors on the lower leg. Case 4 includes sensors on the upper leg and the force plate. Case 5 includes sensors on the lower leg and the force plate. Case 6 includes sensors on the gastrocnemius. Case 7 includes one accelerometer on each segment of the leg. Case 8 includes one accelerometer per shank.

In order to determine which sensors were driving the SVM classification of activities, features that were derived from certain sensors were removed from the total set in a process that is known as ablation [22] (Figure 3). The ablation process serves two purposes: it will help to determine which sensors and sensor placements lead to a higher classification accuracy, and it will lead to a greater understanding regarding which elements of the sensor data gathered drives the classification. The PCA and SVM procedure were performed on the case selected. Comparisons of mean accuracy between cases were evaluated while using Cohen’s *d* effect size in order to consider the differences.

## 3. Results and Discussion

### 3.1. PCA Comparisons

**Figure 4:**
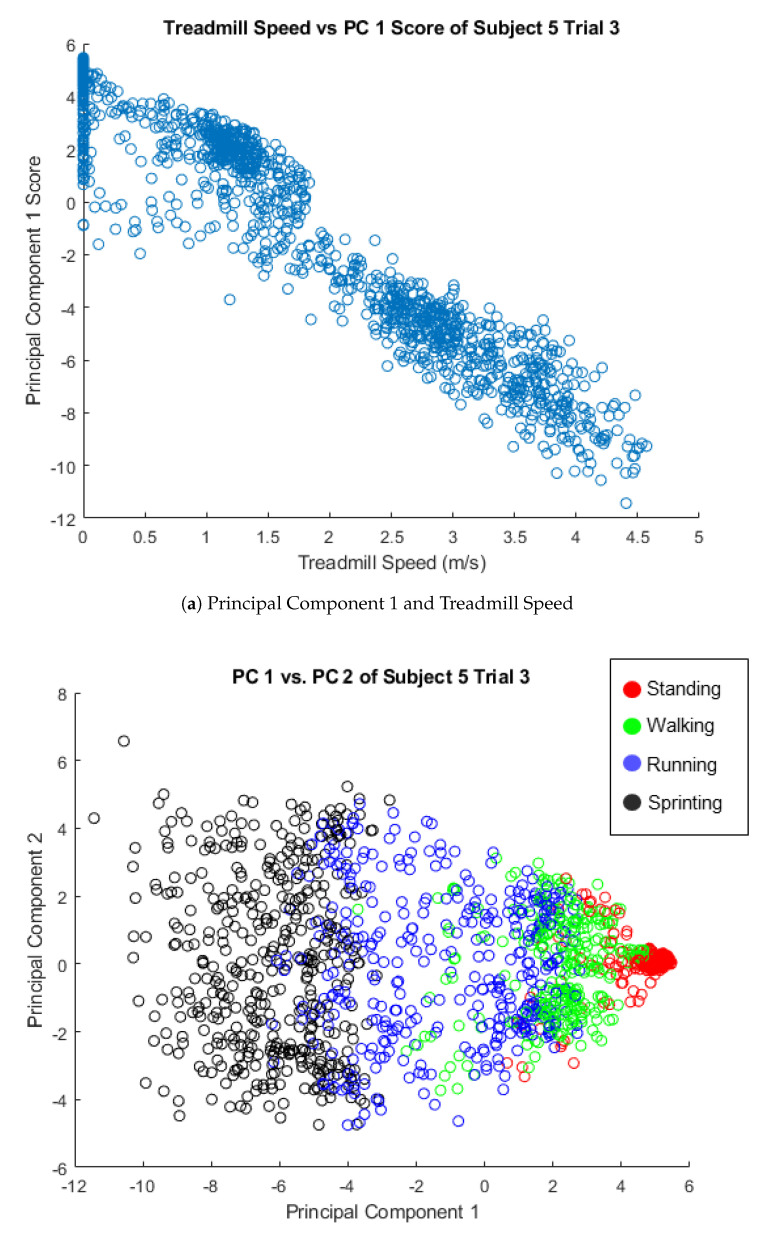
(**a**) A plot of the treadmill speed versus the scores of Principal Component 1. A high level of correlation between these two parameters can be seen with the strong negative slope. This correlation can be seen over all trials, but Subject 5 Trial 3 was selected as a representative case. (**b**) A plot of principal component scores where each data point is colored according to the mode of locomotion the subject was performing. A clear pattern emerges on the Principal Component 1 axis.

**Figure 5:**
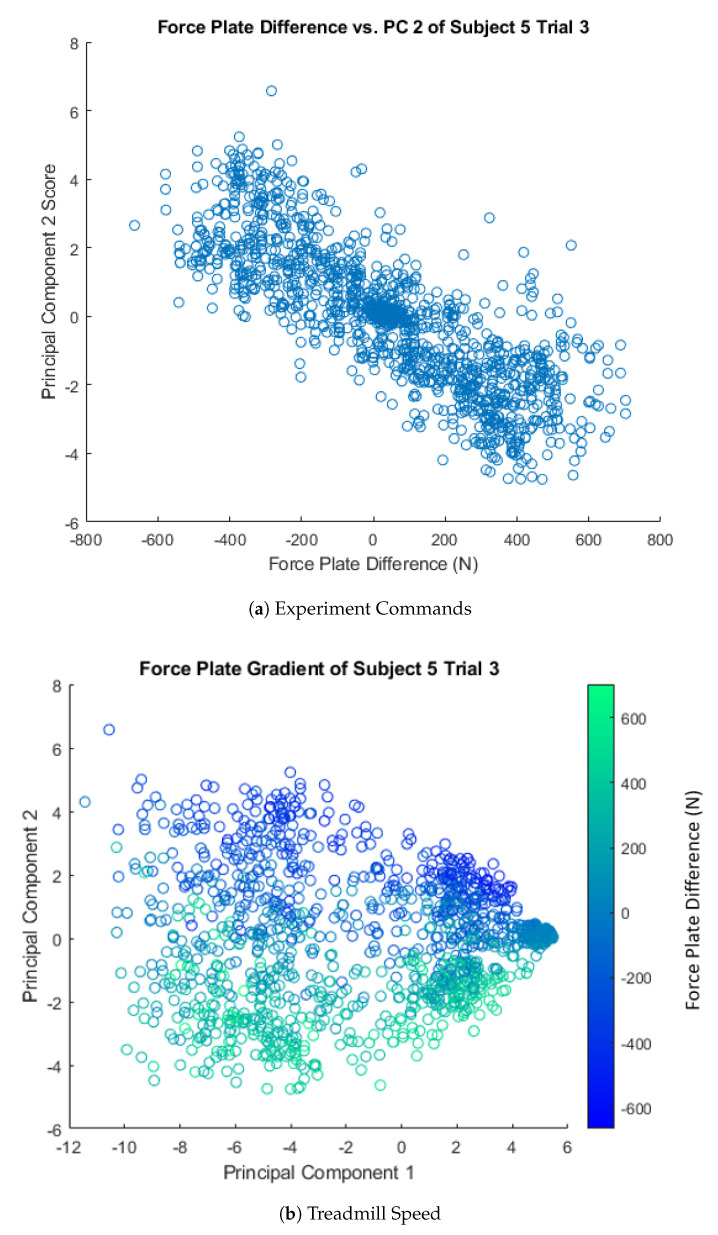
(**a**) A plot of the difference in force plate readings versus the scores of Principal Component 2. A high level of correlation between the two can be seen with the strong negative slope. This correlation can be seen over all trials, but Subject 5 Trial 3 was selected as a representative case. (**b**) Principal component scores plotted with a gradient representing the difference in force plate readings. When the subject places all of their weight on a single leg (single support), the difference between force plates is at the maximum absolute value. The gradient can clearly be seen along the axis of the second principal component, which indicated that this principal component is driven by the variation of right and left leg motion.

Before discussing the comparisons between principal components, we must first understand what these principal components represent. Principal Component 1 was observed to be correlated with treadmill speed, with a median correlation across subjects and trials of ρ=0.96 with an inter-quartile range of ρ=0.02 (Figure 4). The correlations could be positive or negative, depending on the directionality of the principal components of each trial, so the absolute value of each trial’s correlation was taken. Additionally, the type of locomotion was also observed to show distinct clusters across Principal Component 1 (Figure 4). Principal Component 2 of Subjects 2–5 appears to be correlated with the difference between the left and right force plate, which can be used to infer mediolateral positioning. The median correlation value was ρ=0.83 with an inter-quartile range of ρ=0.16 (Figure 5). The difference in force plates readings was observed to show a distinct gradient across the Principal Component 2 axis (Figure 5). Subject 1 did not appear to have a correlation with the force plate data and PC 2, which was likely due to cross-plate strikes, as discussed in more detail in Section 3.4. There was no clear relationship between Principal Component 3 and the collected data observed.

Table 2 presents the median angle between the first three principal components of each trial across subjects and they are individually plotted in Appendix A.

The comparisons of both Components 1 (the first row and the first column) show the smallest angle, 21.05°, is between the like-components, while the other angles are nearly orthogonal. If two sets of principal components were exactly the same, then the diagonal would show 0 degree differences, and all off-diagonal entries would show 90 degree differences. These separation angles support that all first components are similar. The comparisons of Components 2 (the second row and the second column) and of Components 3 (the third row and the third column) have the smallest angle between the like-components as opposed to non-like components. Although the comparisons between the second and third components did not have ideal separations, since the like-components are the smallest, we consider the components that are similar enough to be used for classification, because the like-components are closer to each other than the non-like components. This assumption is further investigated by determining the classification accuracy.

**Figure A5:**
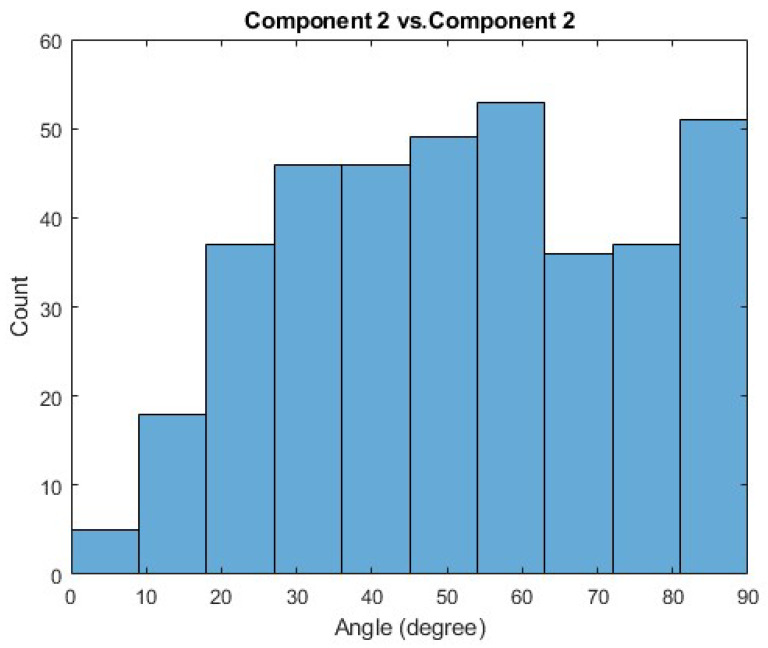
Histogram of the comparisons between the second principal component of some Trial A and the second principal component of some Trial B, where these comparisons are made across all trials and subjects.

The spread observed in comparing Component 2 across trials (Figure A5) may be from the alternation of left and right legs during locomotion, as supported by the correlation of Component 2 and the force plate readings (Figure 5).

### 3.2. Classification Accuracy

**Figure 6:**
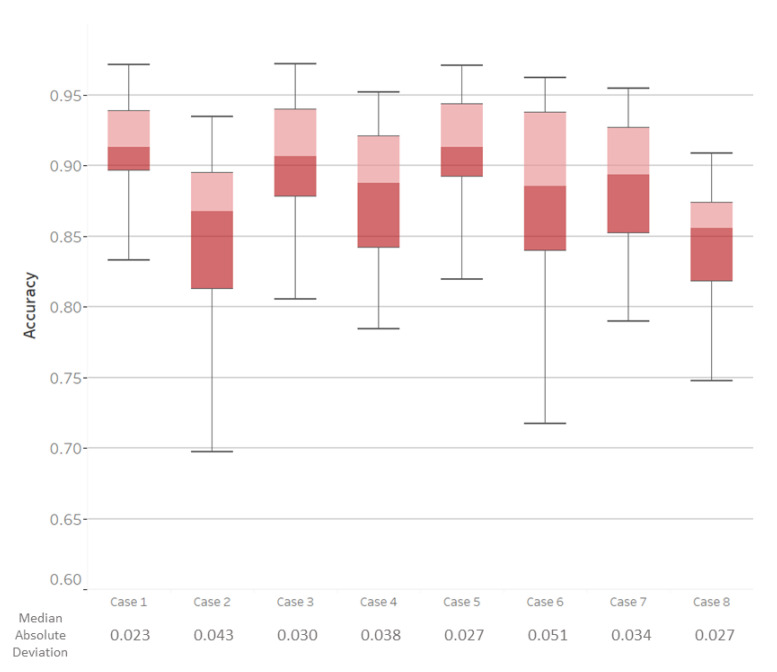
A box plot of the classification accuracy for all eight cases across all subjects with allowed run/sprint confusion. The line on the box represents the median, while the whiskers reach 1.5 times the interquartile range. The cases here refer to the cases that are defined in Figure 3.

The median accuracy across all classes for all sensors (Case 1) was 86.4%, with a median absolute deviation (MAD) of 2.9%. Without allowing for run/sprint confusion, all of the effect sizes for all comparisons between cases were over 0.8 and considered to be large. Consequently, the discussion will only focus on the results when run and sprint confusion is allowed (Table 3). With this confusion, the median accuracy for all sensors was 91.6% with a MAD of 2.3% (Figure 6). A self-paced treadmill allows for subjects to perform locomotion close to their natural gait, but it leads to increased variability in speeds when compared to other studies that use fix-paced treadmills [23]. Despite the increased variability in speeds, a high classification accuracy was obtained with the full suite of sensors and the first three principal components.

The Cohen’s *d* effect sizes across all comparisons that are listed in Table 3 were all large when directly using the predicted labels from the SVM. Only when run/sprint confusion was allowed did the effect sizes range across small, medium, and large, meaning that our reduced sensor sets do not contain enough information to accurately distinguish between running and sprinting. Our subjects were not expert sprinters, so the run and sprint were also similar to each other for this reason.

The results show that a higher level of accuracy is achieved when sensors are on the lower leg (Cases 3 and 5) than when sensors are placed on the upper leg (Cases 2 and 4). Case 3 has a higher accuracy than Case 2 (large effect size), which demonstrates that there is a significant impact on placing sensors on upper versus lower legs. Removing sensors on the lower leg and maintaining sensors on the upper leg, with the force plates (Case 4) as well as without the force plates (Case 2), led to losses in accuracy (large effect size). This loss in accuracy might be because not enough information about the activity is provided from the hamstring and vastus medialis, as they have similar EMG profiles during walking and running [24]. The effect size when both of these cases are compared to Case 1 is large, and Figure 6 shows that the median classification accuracy is lower than in Case 1.

The number of sensors that are placed on the lower leg matters in accurately classifying activities. When the sensors are only on the gastrocnemius muscle (Case 6), there is a decrease in accuracy when compared to Case 1 (large effect size) and a decrease in accuracy when compared to Case 3 (medium effect size). This difference in effect size means that only having two sensors on the lower leg might be insufficient for classification between stand–walk–run. During standing, walking, and running, the gastrocnemius and tibialis anterior co-contract in order to provide stability to the ankle. The removal of the tibialis sEMG sensors eliminates the ability for co-contraction to be captured within the algorithm. These results support the method having a higher accuracy when co-contraction is captured. Additionally, the lower leg is farther away from the center of rotation (the hips), so the differences in gait are magnified in the lower-leg sensors. There is a greater difference in how much the sensors on the lower leg swing during walking and running than the sensors on the upper leg.

Removing the force plates results in changes to accuracy when compared to cases with the force plate. The removal of the force plate led to reductions in accuracy as observed by comparing the sensors on the lower leg, Case 3 vs. Case 5 (medium effect), as well as sensors on the upper leg, Case 2 vs. Case 4 (large effect). The difference here is likely because the magnitude of the normal force is greater during running than walking, which supported the classification between those activities. When compared to the accuracy results from the use of all sensors (Case 1), only using a sensor set on the lower leg with the use of a force plate (Case 5) results in a small effect size (Table 3). If the sensors are limited and the motions of standing, walking, and running need to be classified, utilizing the sensors on the lower leg with force plates may not have operationally relevant differences from the full suite of sensors. When the sensors are only placed on the lower leg and the force plate is not utilized (Case 3), the median accuracy decreases by 0.82% compared to the set with all sensors (Case 1, medium effect size, Table 3). Depending on the use case, this loss in accuracy may or may not be functionally relevant.

Using accelerometers without sEMGs reduces classification accuracy. Using one accelerometer on each segment of the leg (Case 7) decreases the median accuracy when compared to Case 1 (medium effect size). The accelerometer signals provide information on changing speeds, which can inform running and walking. It is likely that the variation in acceleration profiles that comes from using a self-paced system yielded a decrease in accuracy when compared to cases when sEMG signals were present. Case 8 (only four accelerometers) has a decrease in accuracy when compared to Case 1 (large effect size). Even removing two accelerometers when no sEMGs are present yields a large decrease in accuracy, as seen by the large effect size between Cases 7 and 8. When sEMG are not present, the relative motion between the thigh and shank is captured with the accelerometers on these segments. More distal points on the leg experience greater accelerations, as linear acceleration is a function of both the change in angular velocity and the distance from the center of rotation. It follows that the accelerometers on the lower leg capture greater relative motion. These results support that there is a higher accuracy when this segment coordination is captured.

**Figure 7:**
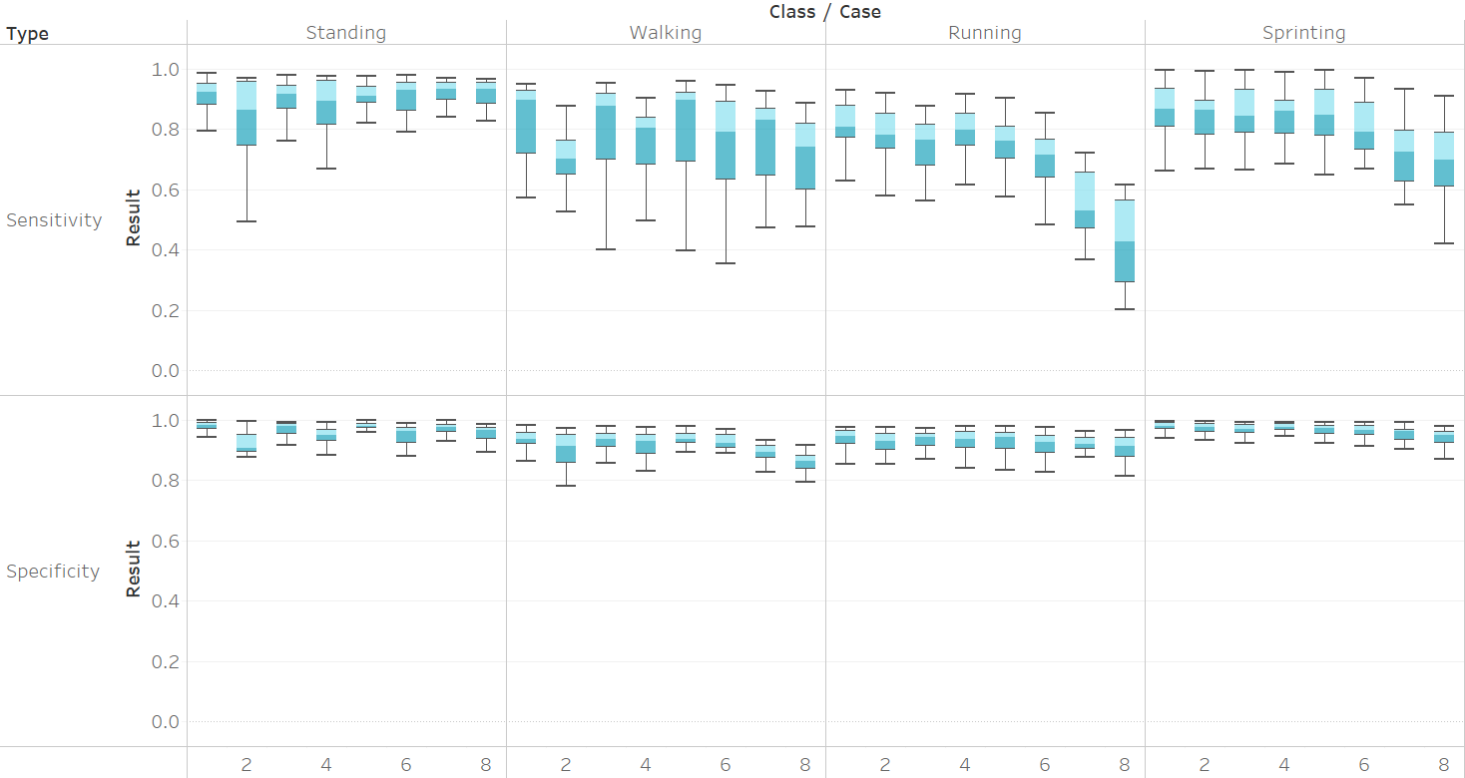
Sensitivities and specificities of each action per case.

Accelerometers alone do not achieve as high accuracies from the SVM as they do when used in tandem with other sensors. In Cases 7 and 8, when only accelerometers were used for classifying all activities, the sensitivity of running sharply decreases (Figure 7). From the lower sensitivity, it can be concluded that the use of accelerometers alone hinders the accuracy of the SVM. Similarly, the walking sensitivity sharply decreases for Case 2, which means that the use of sensors only on the upper leg makes it difficult for a SVM to positively identify walking. Perhaps the lower accuracy in walking is a result of the lowerleg muscles having a greater difference in EMG profiles during running than in walking [24]. Overall, the specificities were much higher than the sensitivities, with most of them being over 90%, which implied that the SVM method of classification does not lead to many false positives in the data, but mostly false negatives. For wearable robotic applications, high specificity might decrease potential harm to subjects. It might be more harmful to misidentify a change in action than perform no change at all, as a misidentificiation might lead to an injury if the robotic system performs in a way that the user is not expecting.

This study extends the literature by examining the effect of sensor choice and placement on classifying standing, walking, and running. Through ablation, we have found that it is best to place sensors on the lower leg. Additionally, the ablation process has helped to gain an understanding regarding how different features contribute to the classification, such as the strong contributions of the lower leg when compared to the upper leg. Although other studies have used PCA for HAR, we have demonstrated that three principal components of this type of data are sufficient for a high degree of classification accuracy.

### 3.3. Applications of the Study

There are many applications to the results of this study. The results here can be used as a heuristic in an exoskeleton controller in order to determine when the wearer shifts from walking to running and vice-versa, supporting different controller needs. For applications, such as fitness tracking, sensors can be placed closer to the ankle than the thigh for the best results, using an EMG if possible. The ablated sensor sets can be used for optimal sensor selection in future studies. The reduced sensor suites and PCA approach can be dimensionally reduced for a faster computation time without compromising accuracy.

This paper demonstrated the successful classification of these data into standing, walking, and running, and enhanced the understanding about which sensor types and placements drive the classification. However, there are opportunities for further exploration. First, other classifiers can be explored. This paper chose to explore the effects of an SVM, which is a type of supervised learning, but there are other supervised learning techniques, such as k-nearest neighbors approximation, which can be utilized. Unsupervised techniques can also be evaluated. Additionally, the techniques that are listed here can be used to classify other types of human activities, and then ablation can be used to study which features drive those classifications.

### 3.4. Limitations of the Study

The small number of subjects was one major limitation of this study. The methods of this paper can be repeated with the use of a greater number of subjects, but, due to global health concerns at the time this paper was written, this was not a possibility for this particular study.

The ability of the subjects to perform the activities as another limitation of the study. Some of the subjects experienced more fatigue than others and slowed during run and sprint, leading to fewer data points for that specific activity to analyze for these cases. Consequently, we have fewer instances of sprinting and running. Because of the space that the study was completed in, we were unable to collect data for other common human activities, such as climbing stairs, but there is opportunity in the future to extend this study to additional activities.

The threshold speed values of each activity were manually set for truth labelling, so some data points might have been mislabelled near transitions. The definition of the truth states was based on the speed that the user was moving at, which was a self-selected process. Occasionally, users transitioned unintentionally. Smoothing the treadmill speeds with a Butterworth filter made the range of speeds for each task more clearly defined, but it did not entirely smooth away every unintentional transition.

The sEMG readings vary from session to session. This study’s data were limited to a single session per subject and, therefore, less variability was encountered in sEMG readings than there would be multi-day study, which would be more similar to a daily use case. It is recommended to collect more data longitudinally in order to assess this variability.

During the study, the subjects were instructed to strike the left force plate with the left foot and the right force plate with the right foot. During analysis, it was observed that cross-plate strikes occurred (e.g., a left foot striking the right force plate), with a greater count towards the end of the study. These cross-strikes may occur, as subjects may have placed less attention on lateral velocity control and foot placement with study duration or fatigue onset. These cross-plate strikes were kept in the dataset. Cross-plate strikes do not affect biomechanical modeling during single-support, but they do affect the modeling of human joint torques during double-support due to the ambiguity in the length of the joints’ moment arm. We do not analyze moments directly in this work, although there would be an effect on our force place sensor inputs.

## 4. Conclusions

This paper demonstrated which sensors were the most important for classifying standing, walking, and running through ablated data sets and an SVM that was trained on principal components. Although the ablated sets did not contain enough information to distinguish between running and sprinting, the results show that using a reduced sensor set on the lower legs will result in a classification accuracy similar to a classification accuracy when all of the sensors are used. Although including the force plates resulted in a higher classification accuracy than when the force plates were not included, the difference might not be operationally relevant. It was also shown that only using accelerometers decreases the sensitivity of the classification algorithm. Our methods involved a greater variation in speeds of activities when compared to using a fixed-speed treadmill, and yet our classification accuracy remained over 90% with all sensors included. This high accuracy indicates that the SVM used on PCA data is an effective tool for HAR and relevant sets of muscles for sensor placements have been found. Moving forward, it would be interesting to learn more regarding why dissimilarities between principal components occur.

## Figures and Tables

**Table 1:** Thresholds of Speeds Used for the Labelling of Each Subject’s Activities.

Speed Threshold (m/s)				
	Stand	Walk	Run	Sprint
Subject 1	0	(0, 1.3]	(1.3, 2.4]	(2.4, ∞]
Subject 2	0	(0, 1.6]	(1.6, 3.3]	(3.3, ∞]
Subject 3	0	(0, 1.6]	(1.6, 2.8]	(2.8, ∞]
Subject 4	0	(0, 1.4]	(1.4, 2.5]	(2.5, ∞]
Subject 5	0	(0, 1.2]	(1.2, 2.7]	(2.7, ∞]

**Table 2:** Median Angle Between Components Across Trial and Subject Combinations.

	Component 1	Component 2	Component 3
Component 1	21.05°	90.65°	89.97°
Component 2	91.15°	51.05°	87.42°
Component 3	90.58°	90.01°	59.71°

**Table 3:** Cohen’s *d* effect sizes.

Comparison	Cohen’s *d* Value- Allowed Run/Sprint Confusion	Effect Size
Case 1 vs. Case 2	1.12	Large
Case 1 vs. Case 3	0.65	Medium
Case 1 vs. Case 4	0.82	Large
Case 1 vs. Case 5	0.29	Small
Case 1 vs. Case 6	0.74	Large
Case 1 vs. Case 7	0.53	Medium
Case 1 vs. Case 8	0.91	Large
Case 7 vs. Case 8	1.66	Large
Case 2 vs. Case 3	−0.85	Large
Case 2 vs. Case 4	−1.04	Large
Case 3 vs. Case 5	−0.67	Medium
Case 3 vs. Case 6	0.65	Medium
Case 6 vs. Case 8	0.52	Medium

**Figure A1:**
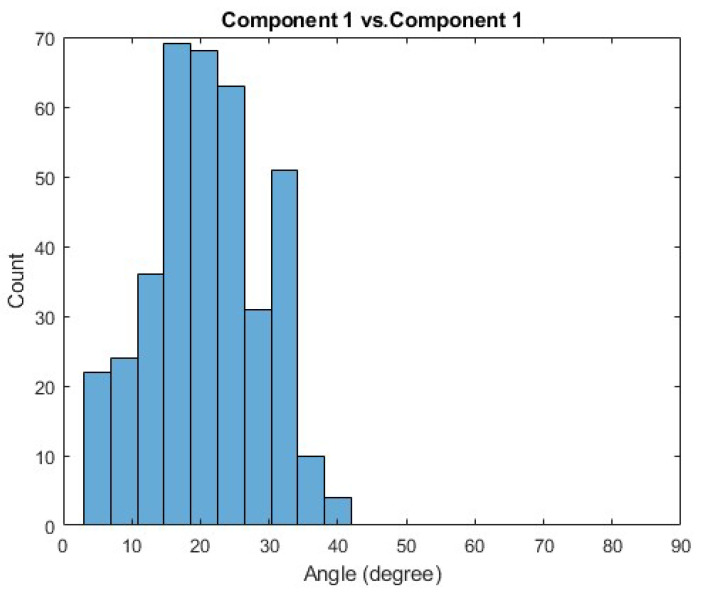
Histogram of the comparisons between the first principal component of some Trial A and the first principal components of some Trial B, where these comparisons are made across all trials and subjects.

**Figure A2:**
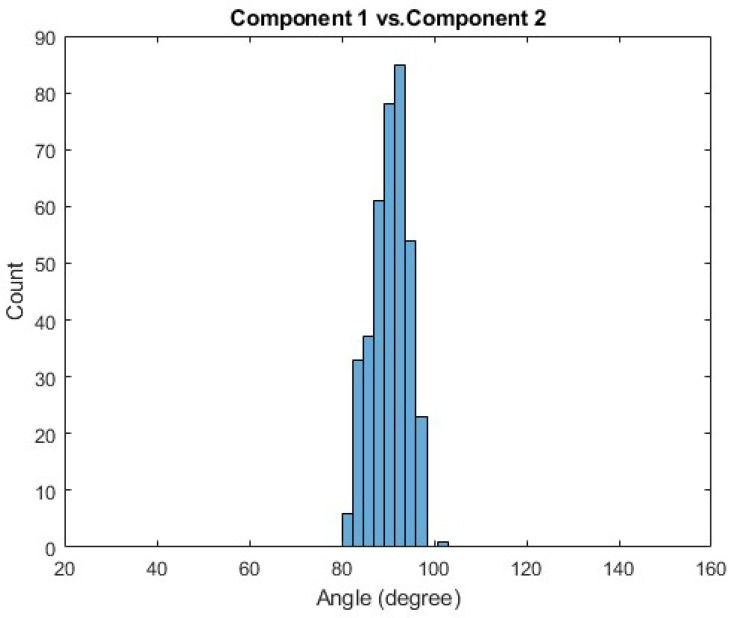
Histogram of the comparisons between the first principal component of some Trial A and the second principal component of some Trial B, where these comparisons are made across all trials and subjects.

**Figure A3:**
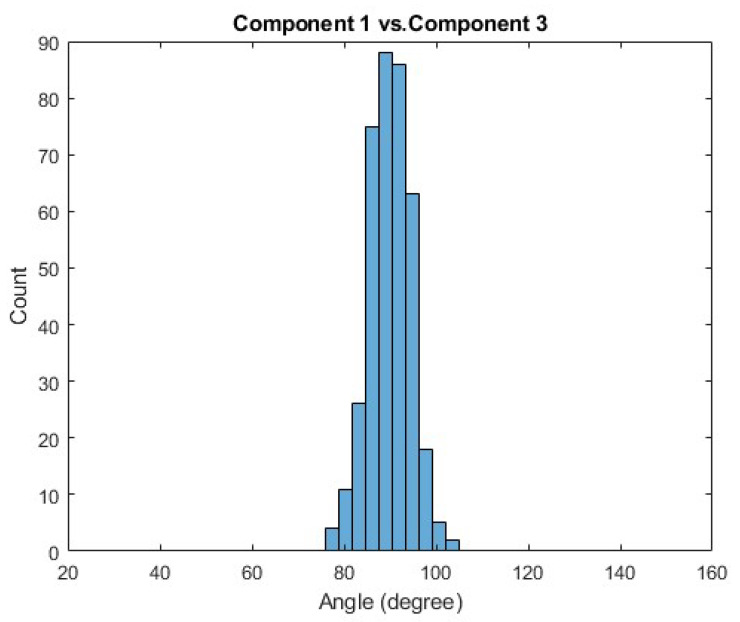
Histogram of the comparisons between the first principal component of some Trial A and the third principal component of some Trial B, where these comparisons are made across all trials and subjects.

**Figure A4:**
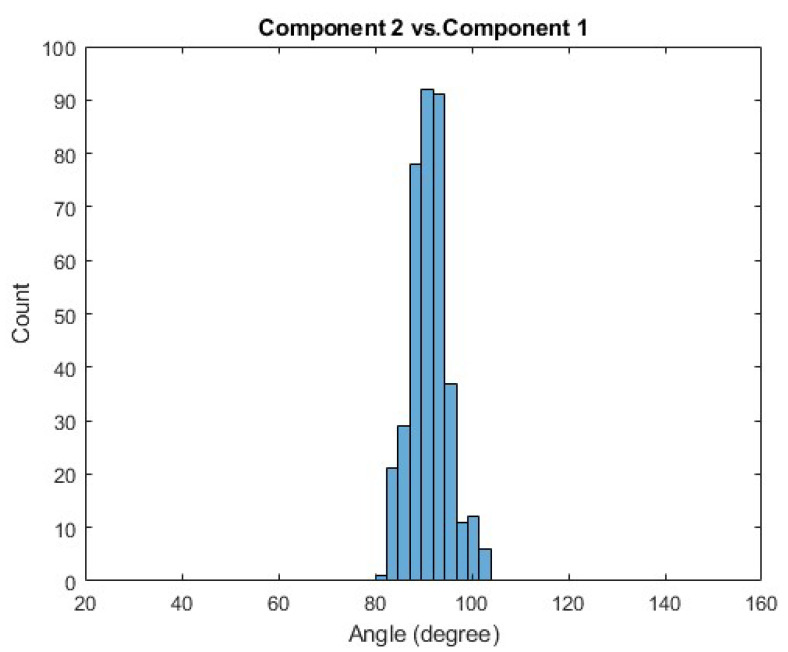
Histogram of the comparisons between the second principal component of some Trial A and the first principal components of some Trial B, where these comparisons are made across all trials and subjects.

**Figure A6:**
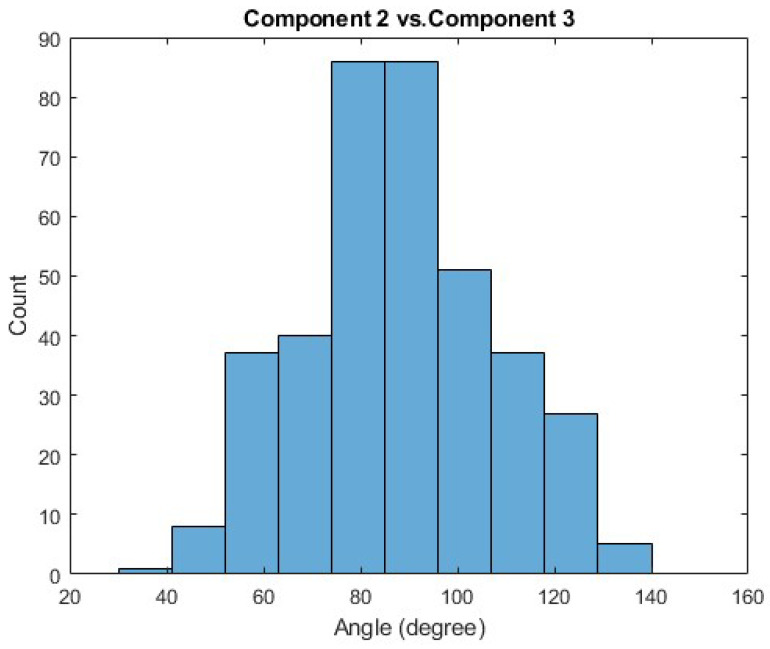
Histogram of the comparisons between the second principal component of some Trial A and the third principal component of some Trial B, where these comparisons are made across all trials and subjects.

**Figure A7:**
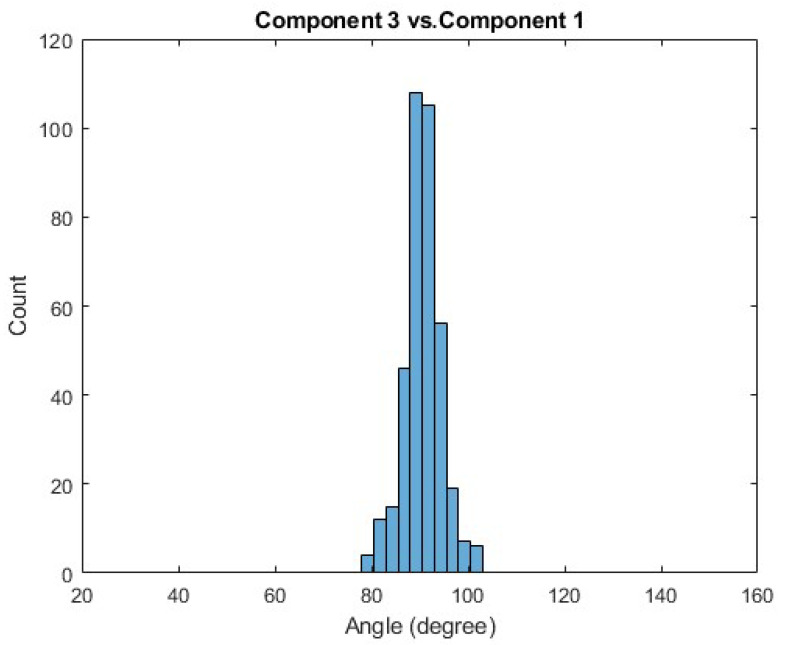
Histogram of the comparisons between the third principal component of some Trial A and the first principal components of some Trial B, where these comparisons are made across all trials and subjects.

**Figure A8:**
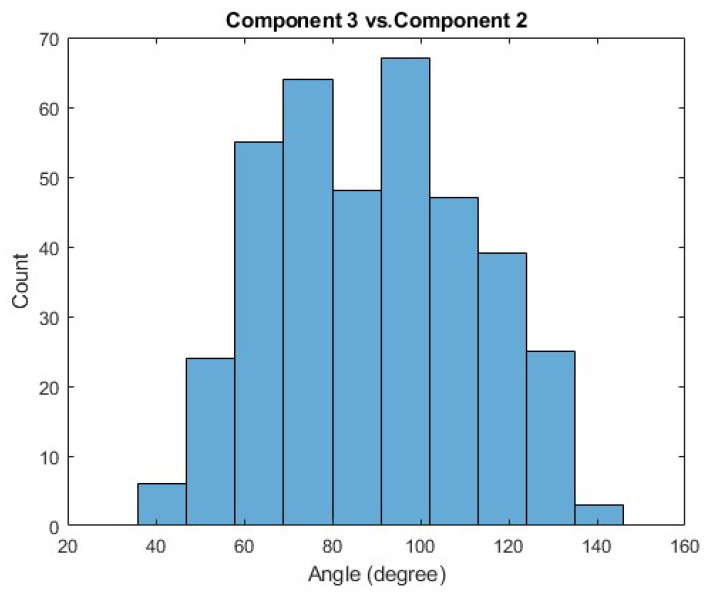
Histogram of the comparisons between the third principal component of some Trial A and the second principal component of some Trial B, where these comparisons are made across all trials and subjects.

**Figure A9:**
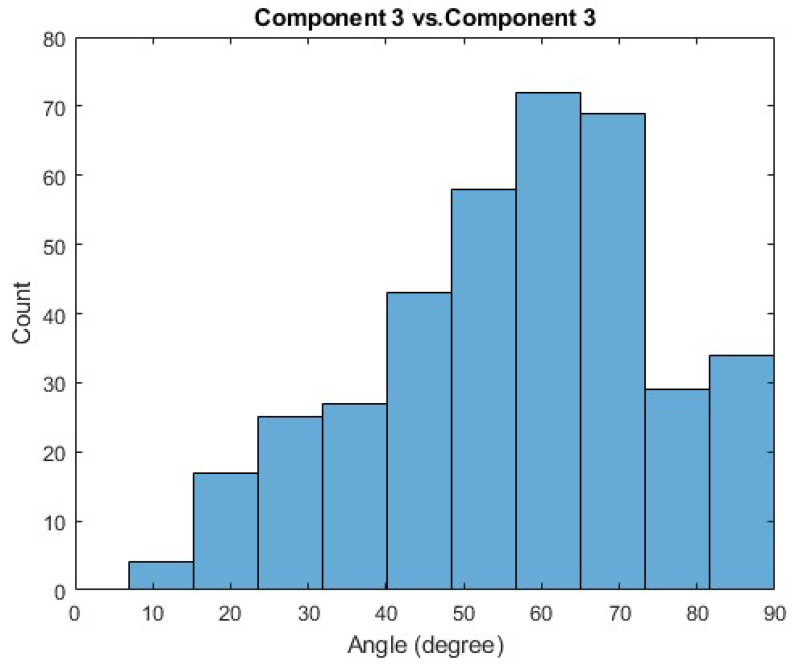
Histogram of the comparisons between the third principal component of some Trial A and the third principal component of some Trial B, where these comparisons are made across all trials and subjects.

## Data Availability

The data presented in this study are available on request from the corresponding author. The data are not publicly available due to continuing study by the authors.

## Appendix A

The following figures are histograms of the angles of separations between all combinations of the principal components.
